# The role of environmental enteric dysfunction in the pathogenesis of *Schistosoma mansoni*-associated morbidity in school-aged children

**DOI:** 10.1371/journal.pntd.0010837

**Published:** 2022-10-05

**Authors:** Jacqueline Araújo Fiuza, Susannah Colt, Letícia Gambogi de Ornellas, Leonardo Ferreira Matoso, Andrea Gazzinelli, Jennifer F. Friedman, Rodrigo Corrêa-Oliveira

**Affiliations:** 1 Grupo de Imunologia Celular e Molecular, Instituto René Rachou, Fundação Oswaldo Cruz, Belo Horizonte, Minas Gerais, Brazil; 2 Center for International Health Research, Rhode Island Hospital, Providence, Rhode Island, United States of America; 3 Department of Pediatrics, The Warren Alpert Medical School of Brown University, Providence, Rhode Island, United States of America; 4 Escola de Enfermagem, Universidade Federal de Minas Gerais, Belo Horizonte, Minas Gerais, Brazil; Centers for Disease Control and Prevention, UNITED STATES

## Abstract

**Background:**

Studies have implicated schistosomiasis as a cause of intestinal barrier disruption, a salient feature of environmental enteric dysfunction (EED), as eggs translocate from the sterile bloodstream through the gut wall. We examined the longitudinal impact of praziquantel (PZQ) treatment on a) EED biomarkers and b) Insulin growth factor I (IGF-1), a key driver of childhood linear growth, since EED has been implicated in linear growth stunting.

**Methodology:**

290 children infected with *S*. *mansoni* in Brazil were treated with PZQ at baseline. EED biomarkers lipopolysaccharide (LPS) and intestinal fatty acid binding-protein (I-FABP) were measured, as well as IGF-1 at baseline, 6 and 12-months. Multivariate regression analysis was applied to assess associations between *S*. *mansoni* intensity and plasma biomarkers (LPS, I-FABP, and IGF-1), controlling for potential confounding variables.

**Principal findings:**

At baseline, *S*. *mansoni* infection intensities were 27.2% light, 46.9% moderate, and 25.9% heavy. LPS concentrations were significantly reduced at the 12-month visit compared to baseline (p = 0.0002). No longitudinal changes were observed for I-FABP or IGF-1 in the 6- or 12-month periods following baseline treatment. After 6-months, I-FABP concentration was significantly higher in high vs low intensity (p = 0.0017). IGF-1 concentrations were significantly lower among children with high and moderate vs low intensity infections at each study visit.

**Conclusions/significance:**

We report that *S*. *mansoni* infection impacts LPS, I-FABP and IGF-1. These findings suggest a mechanistic role for EED in schistosomiasis-related morbidities, particularly linear growth.

## Author summary

*Schistosoma mansoni* is a tropical parasitic infection that causes intestinal schistosomiasis. In infected humans, the parasite worms shed eggs that migrate across the gut barrier, which damages intestinal structure and function. In children, intestinal schistosomiasis leads to anemia, undernutrition, and linear growth stunting. The mechanistic pathways between schistosomiasis and stunting are not fully understood, but this research explores the role of environmental enteric dysfunction (EED) in schistosomiasis-related morbidity. EED is an intestinal condition that affects children living in areas of poor water, sanitation, and hygiene and also leads to impaired growth and stunting. In a longitudinal cohort of Brazilian children infected with *S*. *mansoni*, we measured blood biomarkers of EED and linear growth at three time points over 12 months. All of the children were treated for schistosomiasis at baseline, and after 12 months, we observed a significant decrease in a marker of EED, suggesting improvement in gut integrity. We also found that children who had higher parasite egg burden at the baseline visit had lower levels of insulin-like growth factor-1, a hormone that drives growth in children. Our findings suggest that EED may play a role in schistosomiasis-related stunting and furthers our understanding for *S*. *mansoni* pathogenesis in children.

## Background

Human schistosomiasis, a Neglected Tropical Disease (NTD), affects over 140 million individuals and remains a significant cause of morbidity and mortality in developing countries, despite available drug treatment [1,2]. Two of the three main species affecting humans, *S*. *japonicum* and *S*. *mansoni*, live in the mesenteric venules and deposit thousands of eggs daily that migrate through vessel walls, the interstitium, and ultimately penetrate the gut wall to pass into the gut lumen for excretion in feces. If untreated, higher egg burden and longer duration of infection can lead to more severe morbidity, namely liver fibrosis and hepatosplenic disease, which in some cases culminates in portal hypertension and death [3]. Much of the global burden of disease impacts children, whereby schistosomiasis can cause anemia, undernutrition, linear growth stunting, and impaired neurocognitive development [4–10]. *S*. *mansoni* is endemic in Brazil, one of the few nations in the Americas that has not eliminated this NTD [11,12].

Schistosomiasis is a chronic inflammatory disease, resulting in the elaboration of pro-inflammatory cytokines detectable in the systemic circulation [13–15]. In the context of schistosomiasis, these inflammatory responses are implicated in the pathogenesis of anemia, undernutrition, and linear growth faltering [7,10,13–20]. Prolonged systemic inflammation in children has been linked to disruptions in the insulin-like growth factor (IGF) axis, which is a main regulator of linear growth [21]. Studies in children with chronic inflammatory diseases, both infectious and non-infectious in origin, have shown that increased concentrations of pro-inflammatory biomarkers and decreased concentrations of anabolic growth factors, such as insulin-like growth factor-1 (IGF-1), are associated with linear growth faltering [22–24]. Specific to *S*. *mansoni* infection, children experiencing hepatic fibrosis or hepatosplenic schistosomiasis had significantly lower IGF-1 concentrations compared to uninfected children [25,26].

Although there is a strong correlation between pro-inflammatory cytokines and schistosomiasis-related morbidity [7,14,20], the mechanisms by which schistosome infection results in the elaboration of pro-inflammatory cytokines and consequent morbidity remain understudied. Another driver of inflammatory responses is microbial translocation (MT), whereby luminal microbes cross the intestinal wall into the normally sterile bloodstream [27]. MT is a feature of environmental enteric dysfunction (EED), an acquired subclinical condition of the small intestine associated with impaired linear growth and chronic undernutrition in children exposed to conditions of poor water, sanitation, and hygiene [28,29]. MT is typically detected by the presence of serum lipopolysaccharide (LPS) or endotoxin, LPS binding protein (LBP), or circulating antibodies against LPS [30] in serum. There is increasing evidence linking schistosomiasis with MT. During schistosome infection, the passage of eggs into the gut lumen damages the integrity of the gut wall, which can enable MT into the bloodstream. Schistosome egg-induced MT may represent an important stimulus for pro-inflammatory responses during schistosome infection. Studies have shown higher concentrations of MT biomarkers among adults with schistosomiasis compared to uninfected individuals [31,32]. Further, in adult cases of hepatosplenic schistosomiasis, MT biomarkers were associated with systemic inflammation [33]. However, a recent report among adolescents in Kenya found that LPS was not correlated with schistosome egg burden and was not associated with infection intensity prior to treatment [34].

To our knowledge, studies have yet to examine the impact of schistosomiasis treatment on both gut health markers and IGF-1, a key promotor of linear growth in children. The present study enrolled children infected with *S*. *mansoni* in Brazil to examine a) the relationships between infection intensity (egg burden), gut health markers, and IGF-1 and b) the longitudinal impact of *S*. *mansoni* treatment [praziquantel (PZQ)] on gut health markers and IGF-1. We include gut health markers assessing both MT (LPS) and intestinal epithelial damage [intestinal fatty acid binding-protein (I-FABP)], another characteristic feature of EED.

## Methods

### Ethics statement

This study was approved by the Brazilian National Ethics Committee (CAAE 531.282). For each participant, formal written informed consent was obtained from a parent. Additionally, assent was obtained from participants ≥ 7 years old.

### Study area, population, and design

This longitudinal study was conducted in the Jequitinhonha Valley in northern Minas Gerais State (MG), Brazil. Children with *S*. *mansoni* infection were recruited in schools from multiple communities within five different municipalities between March-December 2014. Participant recruitment and enrollment has been described previously [35]. Students and their parents or legal guardians were invited by members of the study team to participate in the parasitological screening. Students (n = 3,661) provided stool specimens and 20.4% (n = 750) were positive for *S*. *mansoni*. The eligibility criteria included males and females between the ages of 6–15 years with *S*. *mansoni* infection. Those who were pregnant or breastfeeding or who had symptoms of diarrhea were excluded. Enrolled participants attended baseline, 4-week, 6-month, and 12-month study visits. Demographic and socioeconomic information was collected at the baseline visit using questionnaires as previously described [36,37]. The present analysis includes a sample of n = 290 participants who met the inclusion criteria for the cohort study described above and who had available plasma for biomarker measurements (LPS, I-FABP, and IGF-1).

At the baseline visit, all participants received treatment for *S*. *mansoni* infection: a single oral dose of PZQ (60 mg/kg). Any participants with geohelminth infections at baseline were treated with a single oral dose of albendazole (400 mg). Four weeks following PZQ treatment, participants were re-assessed for *S*. *mansoni* infection and retreated if necessary. Re-infection of *S*. *mansoni* was assessed at the 12-month visit, and any positive cases were treated with PZQ. All treatments were administered with direct observation and under medical supervision as recommended by the Brazilian Ministry of Health [38]. At baseline, 6-month, and 12-month visits, blood samples were collected in vacuum blood collection tubes and transported to the laboratory at the Instituto René Rachou, FIOCRUZ for processing, and plasma fractions were stored in -80C freezers. Biomarkers (LPS, I-FABP, and IGF-1) were measured in plasma collected at baseline, 6-month, and 12-month visits.

### Laboratory measures

*S*. *mansoni* infection was determined by microscopy using the Kato-Katz fecal thick smear method [39]. At screening, baseline, 4-week, and 12-month visits, participants provided stool samples collected on two separate days. Slides were prepared and assessed in duplicate for each stool sample within 24 hours of collection. For each visit, the slide concentrations of eggs per gram of stool (EPG) were averaged, and any value equal to or greater than 1 EPG was considered positive. For quality control, 10% of the slides were randomly selected and examined by a senior microscopist at the Instituto René Rachou, FIOCRUZ, in Belo Horizonte, MG. Plasma LPS was quantified using the Pierce LAL Chromogenic Endotoxin Quantitation Kit (Thermo Fisher Scientific, Waltham, MA, USA). Plasma I-FABP and IGF-1 concentrations were measured by enzyme-linked immunosorbent assay (ELISA) using kits from R&D Systems (Human FABP2/I-FABP DuoSet ELISA #DY3078, Human IGF-I/IGF-1 Quantikine ELISA #DG100B). Biomarker measurements were conducted at the Instituto René Rachou, FIOCRUZ in Brazil.

### Statistical analysis

*S*. *mansoni* intensity is reported in eggs per gram of stool (EPG) and intensity categories are defined as light (1–99 EPG), moderate (100–399 EPG), and heavy (≥400 EPG) [39]. Weight-for-age z-score (WAZ) was calculated using the CDC growth chart reference for ages 0 to <20 years, which considers age, weight, and sex. Socioeconomic status (SES) categories were determined using methods designed by Gwatkin, et al. [40] and described previously [35]. Values are reported in median and interquartile range (IQR) unless otherwise stated. Multivariate regression analysis was applied to assess associations between plasma biomarker concentrations and baseline *S*. *mansoni* intensity category and changes in plasma biomarker concentrations across study visits. Fully adjusted models applied automated stepwise selection with entry and stay thresholds of 0.1 to consider potential confounding variables including age, sex, WAZ, SES, and hookworm infection status. Continuous variables included in regression models were natural log-transformed. P values < 0.05 were considered significant. Statistical analyses were conducted using SAS Studio 3.8 (SAS Institute Inc., Cary, NC).

## Results

### Population

Characteristics for the 290 participants are described in Table 1. The median age was 12.2 years (10.0–13.8 IQR), and 57.2% were male. The median *S*. *mansoni* intensity was 192 EPG (90–516 IQR), and the proportions in light, moderate, and heavy intensity categories were 27.2%, 46.9%, and 25.9%, respectively. Of the 290 participants treated for *S*. *mansoni* infection at baseline, 46 (16.2%) were re-infected at the 12-month visit. Analyses pertaining to the 12-month visit were also run separately excluding those 46 participants who were re-infected, and none of the significant findings were impacted.

**Table 1:** Participant characteristics at baseline, N = 290

	n (%) or median (IQR)
**Age (years)**	12.2 (10.0–13.8)
**Sex (male)**	166 (57.2)
**Weight (Kg)**	36.0 (29.0–47.9)
**Weight-For-Age z-score (WAZ)**	-0.55 (-1.27, 0.21)
**Socioeconomic status (SES) level**	
1 (Lowest)	83 (29.3)
2	47 (16.6)
3	70 (24.7)
4	83 (29.3)
5 (Highest)	0 (0)
**Community site**	
Astraluta	18 (6.2)
Caju	30 (10.3)
Córrego São João	40 (13.8)
Giru	38 (13.1)
Itaobim	21 (7.2)
Monte Formoso	39 (13.4)
Palha	26 (9.0)
Ponto dos Volantes	78 (26.9)
***S*. *mansoni* intensity (EPG)**	192 (90–516)
***S*. *mansoni* intensity Category**	
Light (1–99 EPG)	79 (27.2)
Moderate (100–399 EPG)	136 (46.9)
Heavy (≥400 EPG)	75 (25.9)
**Hookworm infection status**	32 (11.3)
**Biomarkers**	
LPS (EU/mL)	0.26 (0.20–0.31)
I-FABP (pg/mL)	524.7 (283.3–865.5)
IGF-1 (pg/mL)	1,674.6 (969.4–2,259.7)

EPG, eggs per gram of stool; LPS, lipopolysaccharide; I-FABP, intestinal fatty acid binding protein; IGF-1, insulin-like growth factor 1

### LPS

**Fig 1:**
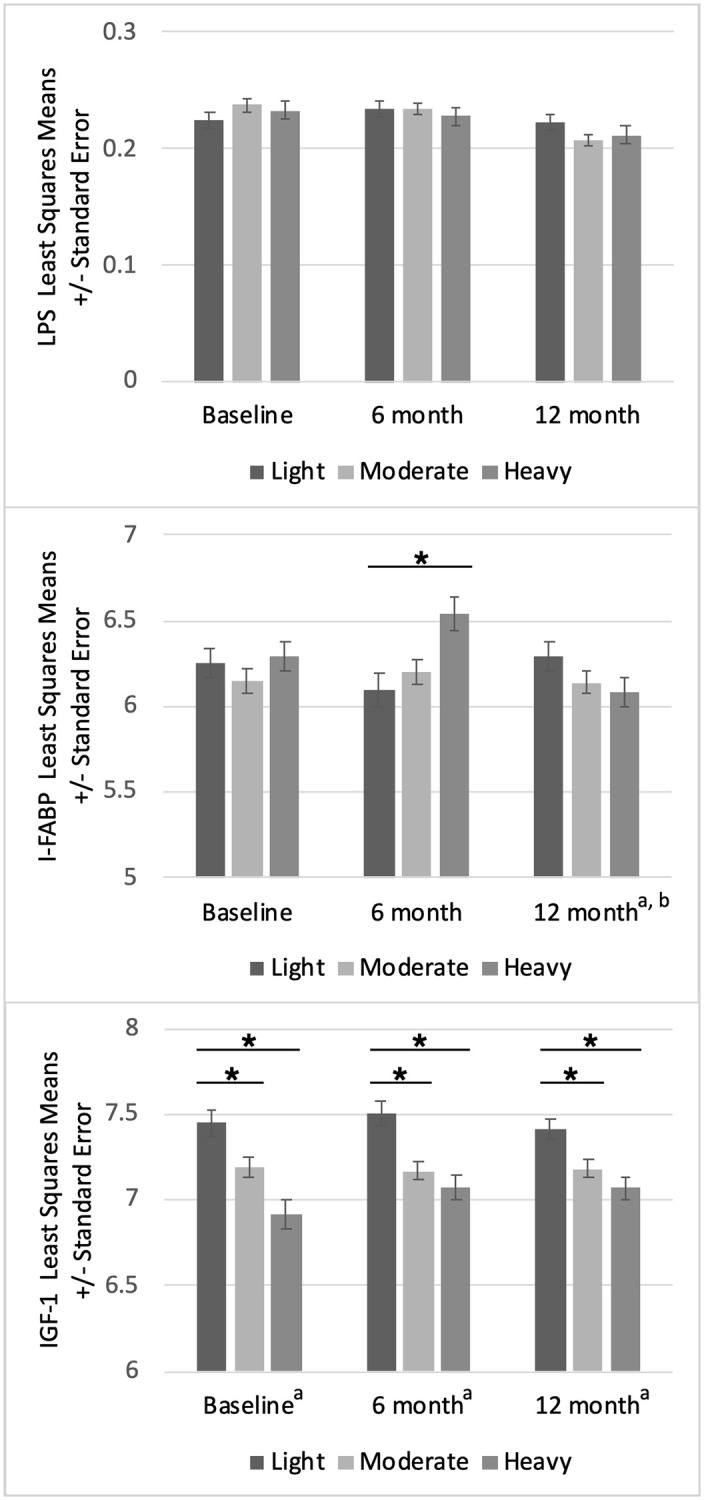
Biomarkers by baseline *S*. *mansoni* intensity. Least squares means and standard errors for lipopolysaccharide (LPS, EU/mL), intestinal fatty acid binding protein (I-FABP, pg/mL), and insulin-like growth factor 1 (IGF-1, pg/mL) concentrations at each visit (baseline, 6-months, and 12-months) by baseline *S*. *mansoni* intensity category. Asterisks represent significant differences determined by multivariate regression with light infection intensity as the reference category. Stepwise selection retained variables of a) age and b) WAZ. Continuous variables were natural log-transformed.

**Fig 2:**
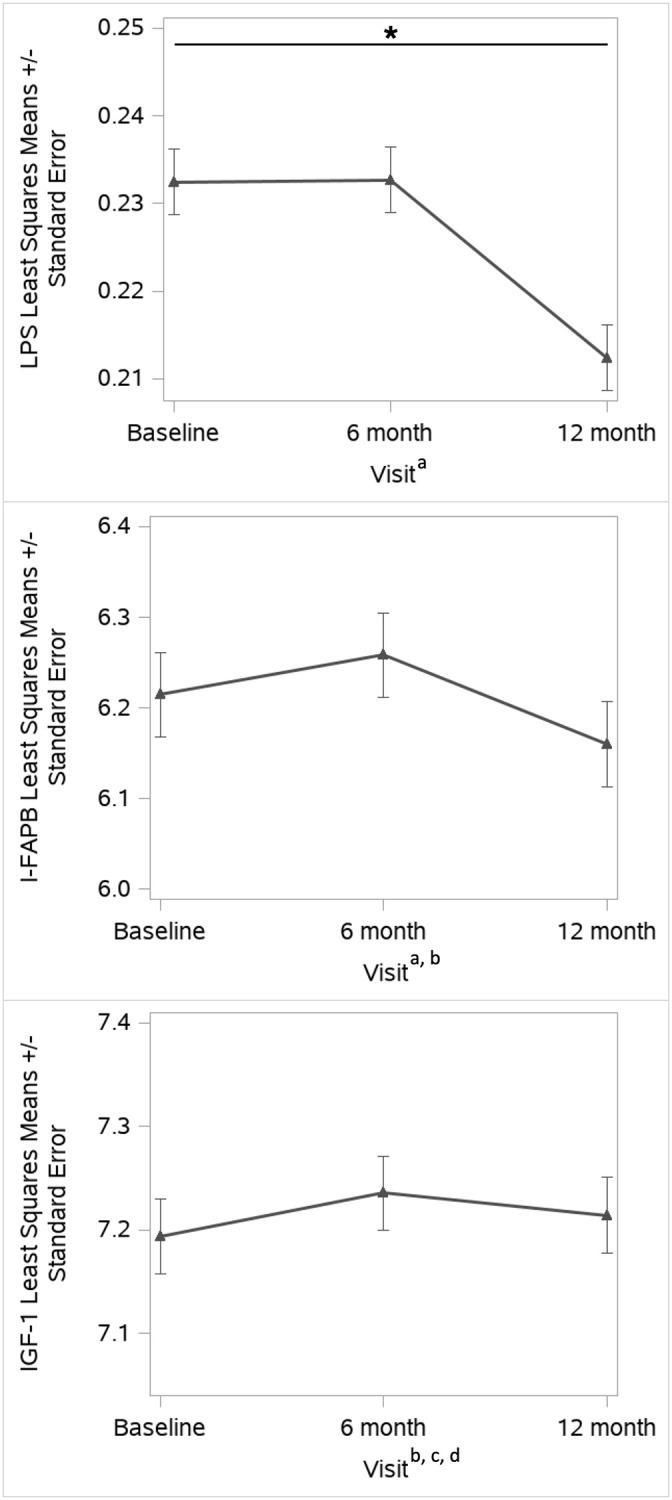
Biomarkers across visits. Least squares means and standard errors for lipopolysaccharide (LPS, EU/mL), intestinal fatty acid binding protein (I-FABP, pg/mL), and insulin-like growth factor 1 (IGF-1, pg/mL) concentrations across visits (baseline, 6-months, and 12-months). Asterisks represent significant differences determined by multivariate regression with the baseline visit as the reference category. Stepwise selection retained variables of a) WAZ, b) age, c) sex, and d) *S*. *mansoni* infection intensity category. Continuous variables were natural log-transformed.

LPS concentration did not differ by *S*. *mansoni* intensity category at baseline nor at the 6- and 12-month follow-up visits (Fig 1, S1 Table). Following PZQ treatment administered during the baseline visit, LPS concentrations were significantly reduced at the 12-month visit compared to the baseline (Fig 2, S2 Table**)**.

### I-FABP

There were no differences in I-FABP concentrations by infection intensity category at the baseline or 12-month visits. At the 6-month visit, the I-FABP concentration was significantly higher for those in the heavy intensity category compared to the light intensity category (Fig 1, S1 Table). I-FABP concentrations measured at the 6 and 12 month follow-up visits were not significantly different from the baseline measures (Fig 2, S2 Table).

### IGF-1

IGF-1 concentrations were significantly lower for those with moderate and heavy infection intensity compared to light infection intensity at baseline, 6-month, and 12-month visits (Fig 1, S1 Table). There were no significant differences in IGF-1 concentrations measured at the 6 and 12-month visits compared to the baseline visit **(**Fig 2, S2 Table). In multivariate regression models adjusting for age, IGF-1 concentrations were positively correlated with LPS concentrations at the 6-month (p = 0.0218) and 12- month visits (p = 0.0445), but not at baseline (p = 0.1105). In similar regression models adjusting for age, IGF-1 concentrations were not correlated with I-FABP concentrations at any visit (Table 2).

**Table 2:** Linear regression of IGF-1 concentrations by EED biomarkers

	Univariate		Multivariate	
	ß	P value	ß	P value^a^
**LPS (EU/mL)**				
Baseline	1.0032	0.1399	1.0526	0.1105
6-month	1.4388	**0.0248**	1.4454	**0.0218**
12-month	1.0588	**0.0497**	1.0650	**0.0445**
**I-FABP (pg/mL)**				
Baseline	-0.0544	0.3552	-0.0196	0.7331
6-month	-0.0914	**0.0387**	-0.0685	0.1200
12-month	-0.0272	0.5464	-0.0078	0.8620

LPS, lipopolysaccharide; I-FABP, intestinal fatty acid binding protein; IGF-1, insulin-like growth factor 1. Continuous variables were natural log-transformed.

All continuous variables natural log-transformed.

^a)^ Stepwise selection considered variables of age, sex, WAZ, SES, and hookworm infection status. Of these, only age was retained and included in all multivariate regression models.

## Discussion

To better understand the mechanistic pathway between childhood schistosomiasis and associated morbidities, such as impaired linear growth, this longitudinal cohort study sought to examine the relationships between *S*. *mansoni* infection and markers of gut health and the IGF axis as one potential pathway. The results demonstrate that *S*. *mansoni* infection impacts EED biomarkers, LPS and I-FABP, as well as IGF-1.

Compared to light infection intensity at baseline, participants with heavy or moderate infection intensity had significantly lower concentrations of IGF-1 measured at baseline, 6-month, and 12-month visits. These age-adjusted findings contribute evidence that schistosomiasis egg burden is associated with disruptions in the IGF axis, with long-term impacts during the 12 months following PZQ treatment. While the evidence is limited, previous case-control studies have demonstrated associations between severe forms of disease–hepatic fibrosis or hepatosplenic schistosomiasis–and decreased IGF-1 concentrations compared to control groups [25,26]. However, this is the first study to demonstrate an impact of schistosome egg burden on IGF-1 in the absence of overt clinical disease. These findings suggest that intensity of schistosomiasis infection leads to disruption in the IGF axis, which contributes to our understanding of at least one mechanism underlying schistosomiasis-related morbidities, specifically impaired linear growth in children.

There is strong evidence to support that chronic pro-inflammatory responses result in impaired linear growth for children experiencing schistosomiasis or EED [14,21,23]. Cytokines characteristic of a pro-inflammatory or T helper type-1 (Th1) mediated response disrupt the IGF axis and culminate in impaired growth. This has been demonstrated in chronic inflammatory childhood diseases, such as inflammatory bowel disease and chronic kidney disease, whereby increased inflammatory biomarkers and decreased anabolic growth factors like IGF-1 are associated with linear growth faltering, as well as in insults of bacteremia from MT [21,24]. Previous studies have shown that the MT biomarker LPS negatively impacts IGF-1 [41,42], however, in this cohort, LPS concentrations were positively correlated with IGF-1 six and 12 months after PZQ treatment, and not at baseline during active infection. In other studies, a negative relationship between LPS and IGF-1 has been shown to be mediated by polarized Th1/Th2 responses [43,44], and additional measures of associated cytokines would offer a more comprehensive understanding of the MT and IGF axis associations in this population. Given that schistosomiasis is a chronic inflammatory disease affecting children, future research ought to target the mechanistic roles of inflammation and the IGF axis as they relate to childhood morbidities, such as growth faltering.

Our findings demonstrate the impact of *S*. *mansoni* infection on LPS. In adjusted regression models, LPS concentrations were significantly lower 12 months following PZQ treatment compared to baseline. Similar studies evaluating MT biomarkers following PZQ treatment for schistosomiasis showed no significant impact on LPS 6 months or 9 months after treatment [45,46]. Our results suggest an even longer timeframe required for gut barrier healing following the cessation of schistosome eggs traversing the gut wall.

The EED biomarker I-FABP is an intracellular enterocyte protein released into circulation following injury to the gut epithelia [47,48]. To our knowledge, only one previous study has examined I-FABP in the context of schistosomiasis [49]. In that study, adult fishermen in Kenya with HIV infection, and some of whom were infected with *S*. *mansoni*, were treated with antiretroviral therapy and PZQ at baseline as needed. I-FABP was measured at baseline, 2 weeks, 1 month, and 3 months post treatment. They found that I-FABP concentrations among participants with *S*. *mansoni* infection did not differ between visits and were not associated with *S*. *mansoni* EPG at any visit. However, those cases had relatively low infection intensity compared to the current analysis. We observed associations between infection intensity category and I-FABP concentrations 6 months following PZQ treatment. Concentrations of I-FABP were significantly higher for participants with high egg burden compared to low egg burden. Higher I-FABP concentrations related to higher egg burden at the 6-month visit may be explained by residual eggs lodged in the tissue between the mesentery and the luminal barrier and associated granulomatous inflammation, requiring time to more fully resolve [50,51].

Because 16.2% of participants were found to be re-infected with *S*. *mansoni* infection at the 12-month follow-up visit, we re-ran the regression models pertaining to the 12-month visit and excluded any participants who had become re-infected. Excluding these re-infected participants had no impact on the significant findings reported in the results section, and no additional significant associations emerged.

The present findings offer evidence to support relationships between *S*. *mansoni* infection and both EED and the IGF axis and further our understanding of the mechanistic pathways between schistosomiasis and associated morbidities. The analysis of these mechanisms would be strengthened by measuring circulating immunologic response markers (Th1 and Th2 cytokines) at each visit as well as collecting measures on schistosomiasis-specific morbidities (anemia, linear growth, and hepatic fibrosis). Additionally, the findings would benefit from enrolling participants without baseline *S*. *mansoni* infection who represent similar geographic and demographic characteristics. Given the significant overlapping global burden of schistosomiasis and EED, it is essential that we elucidate the immunopathology of these illnesses in order to prevent long-term morbidities in affected populations.

## Supporting information

S1 File STROBE Statement. Checklist of items that should be included in reports of cohort studies.
(DOCX)

S1 Table Linear regression of biomarkers by baseline *S*. *mansoni* infection intensity category. These findings are reflected in Fig 1. All continuous variables natural log-transformed. Stepwise selection variables for multivariate regression included a) age, b) weight-for-age z-score. LPS, lipopolysaccharide; I-FABP, intestinal fatty acid binding protein; IGF-1, insulin-like growth factor 1.
(DOCX)

S2 Table Linear regression of biomarkers by study visit. These findings are reflected in Fig 2. All continuous variables natural log-transformed. Stepwise selection variables for multivariate regression included a) weight-for-age z-score, b) age, c) sex, d) *S*. *mansoni* intensity category. LPS, lipopolysaccharide; I-FABP, intestinal fatty acid binding protein; IGF-1, insulin-like growth factor 1.
(DOCX)
