# Supplementary material for: The role of environmental enteric dysfunction in the pathogenesis of Schistosoma mansoni-associated morbidity in school-aged children
Source: PLoS Negl Trop Dis. 2022 Oct 5;16(10):e0010837. doi: 10.1371/journal.pntd.0010837 (PMC9576041; doi:10.1371/journal.pntd.0010837)
Supplement: S1 Table — These findings are reflected in Fig 1. All continuous variables natural log-transformed. Stepwise selection variables for multivariate regression included a) age, b) weight-for-age z-score. LPS, lipopolysaccharide; I-FABP, intestinal fatty acid binding protein; IGF-1, insulin-like growth factor 1. (DOCX) [file pntd.0010837.s002.docx]

**S1 Table. Linear regression of biomarkers by baseline *S. mansoni* infection intensity category.**

|  | **Univariate** | | | | **Multivariate** | | | |
| --- | --- | --- | --- | --- | --- | --- | --- | --- |
| **Intensity** | **Moderate vs Low** | | **High vs Low** | | **Moderate vs Low** | | **High vs Low** | |
| **LPS (EU/mL)** | **ß** | **P value** | **ß** | **P value** | **ß** | **P value** | **ß** | **P value** |
| Baseline | 0.0130 | 0.1630 | 0.0080 | 0.4494 | 0.0130 | 0.1630 | 0.0080 | 0.4494 |
| 6-month | -0.00004 | 0.9966 | -0.0066 | 0.5248 | -0.00004 | 0.9966 | -0.0066 | 0.5248 |
| 12-month | -0.0161 | 0.0669 | -0.0113 | 0.2606 | -0.0161 | 0.0669 | -0.0113 | 0.2606 |
| **I-FABP (pg/mL)** |  |  |  |  |  |  |  |  |
| Baseline | -0.1033 | 0.3486 | 0.0377 | 0.7618 | -0.1033 | 0.3486 | 0.0377 | 0.7618 |
| 6-month | 0.1011 | 0.4051 | 0.4388 | **0.0017** | 0.1011 | 0.4051 | 0.4388 | **0.0017** |
| 12-month ^a, b^ | -0.1072 | 0.3353 | -0.1199 | 0.3374 | -0.1550 | 0.1545 | -0.2159 | 0.0792 |
| **IGF-1 (pg/mL)** |  |  |  |  |  |  |  |  |
| Baseline ^a^ | -0.2970 | **0.0033** | -0.5932 | **<0.0001** | -0.2531 | **0.0107** | -0.5313 | **<0.0001** |
| 6-month ^a^ | -0.3670 | **<0.0001** | -0.4793 | **<0.0001** | -0.3372 | **0.0001** | -0.4334 | **<0.0001** |
| 12-month ^a^ | -0.2525 | **0.0013** | -0.3777 | **<0.0001** | -0.2286 | **0.0033** | -0.3431 | **0.0001** |

These findings are reflected in Fig 1.

All continuous variables natural log-transformed.

Stepwise selection variables for multivariate regression included a) age, b) weight-for-age z-score.

LPS, lipopolysaccharide; I-FABP, intestinal fatty acid binding protein; IGF-1, insulin-like growth factor 1.
