# Supplementary material for: The role of environmental enteric dysfunction in the pathogenesis of Schistosoma mansoni-associated morbidity in school-aged children
Source: PLoS Negl Trop Dis. 2022 Oct 5;16(10):e0010837. doi: 10.1371/journal.pntd.0010837 (PMC9576041; doi:10.1371/journal.pntd.0010837)
Supplement: S2 Table — These findings are reflected in Fig 2. All continuous variables natural log-transformed. Stepwise selection variables for multivariate regression included a) weight-for-age z-score, b) age, c) sex, d) S. mansoni intensity category. LPS, lipopolysaccharide; I-FABP, intestinal fatty acid binding protein; IGF-1, insulin-like growth factor 1. (DOCX) [file pntd.0010837.s003.docx]

**S2 Table. Linear regression of biomarkers by study visit.**

|  | **Univariate** | | | | **Multivariate** | | | |
| --- | --- | --- | --- | --- | --- | --- | --- | --- |
|  | **6-month vs Baseline** | | **12-month vs Baseline** | | **6-month vs Baseline** | | **12-month vs Baseline** | |
|  | **ß** | **P value** | **ß** | **P value** | **ß** | **P value** | **ß** | **P value** |
| **LPS (EU/mL)**^a^ | 0.0002 | 0.9626 | -0.0200 | **0.0002** | 0.0002 | 0.9625 | -0.0200 | **0.0002** |
| **I-FABP (pg/mL)** ^a, b^ | 0.0443 | 0.5082 | -0.0525 | 0.4391 | 0.0436 | 0.5066 | -0.0549 | 0.4084 |
| **IGF-1 (pg/mL)** ^b, c, d^ | 0.0462 | 0.3951 | 0.0292 | 0.5954 | 0.0418 | 0.4135 | 0.0202 | 0.6961 |

These findings are reflected in Fig 2.

All continuous variables natural log-transformed.

Stepwise selection variables for multivariate regression included a) weight-for-age z-score, b) age, c) sex, d) S. mansoni intensity category.

LPS, lipopolysaccharide; I-FABP, intestinal fatty acid binding protein; IGF-1, insulin-like growth factor 1.
